# The diagnostic significance of integrating m6A modification and immune microenvironment features based on bioinformatic investigation in aortic dissection

**DOI:** 10.3389/fcvm.2022.948002

**Published:** 2022-08-29

**Authors:** Ruiming Guo, Jia Dai, Hao Xu, Suhua Zang, Liang Zhang, Ning Ma, Xin Zhang, Lixuan Zhao, Hong Luo, Donghai Liu, Jian Zhang

**Affiliations:** Department of Cardiovascular Surgery, The First Affiliated Hospital of Zhengzhou University, Zhengzhou, China

**Keywords:** m6A, aortic dissection, immune microenvironment, diagnosis, bioinformatic investigation

## Abstract

**Purpose:**

The aim of this study was to investigate the role of m6A modification and the immune microenvironment (IME) features in aortic dissection (AD) and establish a clinical diagnostic model for AD based on m6A and IME factors.

**Methods:**

GSE52093, GSE98770, GSE147026, GSE153434, and GSE107844 datasets were downloaded from the GEO database. The expression of 21 m6A genes including m6A writers, erasers, readers, and immune cell infiltrates was analyzed in AD and healthy samples by differential analysis and ssGSEA method, respectively. Both correlation analyses between m6A genes and immune cells were conducted by Pearson and Spearman analysis. XGboost was used to dissect the major m6A genes with significant influences on AD. AD samples were classified into two subgroups *via* consensus cluster and principal component analysis (PCA) analysis, respectively. Among each subgroup, paramount IME features were evaluated. Random forest (RF) was used to figure out key genes from AD and healthy shared differentially expressed genes (DEGs) and two AD subgroups after gene ontology (GO) and Kyoto Encyclopedia of Genes and Genomes (KEGG) analysis. Finally, we constructed an AD diagnostic model combining important m6A regulatory genes and assessed its efficacy.

**Results:**

Among 21 m6A genes, WTAP, HNRNPC, and FTO were upregulated in AD samples, while IGF2BP1 was downregulated compared with healthy samples. Immune cell infiltrating analysis revealed that YTHDF1 was positively correlated with γδT cell level, while FTO was negatively correlated with activated CD4+ T cell abundance. FTO and IGF2BP1 were identified to be crucial genes that facilitate AD development according to the XGboost algorithm. Notably, patients with AD could be classified into two subgroups among which 21 m6A gene expression profiles and IME features differ from each other *via* consensus cluster analysis. The RF identified SYNC and MAPK1IP1L as the crucial genes from common 657 shared common genes in 1,141 DEGs between high and low m6A scores of AD groups. Interestingly, the AD diagnostic model coordinating SYNC and MAPK1IP1L with FTO and IGF2BP1 performed well in distinguishing AD samples.

**Conclusion:**

This study indicated that FTO and IGF2BP1 were involved in the IME of AD. Integrating FTO and IGF2BP1 and MAPK1IP1L key genes in AD with a high m6A level context would provide clues for forthcoming AD diagnosis and therapy.

## Introduction

Aortic dissection (AD) is a life-threatening disease that is responsible for a large percentage of aortic-related deaths (1, 2). Recent studies have discovered that there exist reciprocal interactions among several pathological processes such as dyslipidemia, hypertension, vascular inflammation, and especially autoimmune and infectious diseases, which are engaged in aggravating the development and progression of AD (3). Sustained aortic inflammation and alterations in the level of elasticity of the aortic media considerably elevate the risk of intimal destruction (4). Besides, the rewiring of the arterial wall is modulated by the immune-inflammatory mechanisms, demonstrating the complicated crosstalk between immune cells and inflammatory factors in the evolution of AD (5–7). AD, whether caused by inherited disorders involving connective tissue or by intrinsic instability of the aortic wall, tends to be associated with a poor prognosis. Immediate diagnostic confirmation and effective therapeutic strategies are indispensable for managing affected patients.

The formation and development of AD is a cascade of complex pathological processes modulated by both genetic and epigenetic mechanisms (8, 9). Considering that genetics is not available to fully elucidate its impact on patients, epigenetics in AD is increasingly emerging as an integral factor in deciphering the landscape and context of AD. RNA modification, present across all kingdoms of life, has been considered the third layer of epigenetics, administering the production and metabolism of RNA. Recently, more than 150 modification patterns have been identified, including 5-methylcytosine (m5C), N1-methyladenosine (m1A), and N6-methyladenosine (m6A), with m6A appearing to account for the majority of RNA modifications (10). The processes and biological functions of m6A modification are exclusively handled by methyltransferases (also named m6A writers as follows: METTL3, METTL14, WTAP, ZC3H13, and RBM15/15B), demethylases (also named m6A erasers as follows: ALKBH5 and FTO), and binding proteins (also named m6A readers: YTHDC1/2, YTHDF1/2/3, CBLL1, HNRNPC, HNRNPA2B1, FMR1, LRPPRC, RBMX, ELAV1, and IGF2BP1) (8, 11–13).

Expanding literature has validated that the m6A modification can partly decode intrinsic regulatory mechanisms underlying immune modulation. For instance, YTHDF1 governs enduring neoantigen-specific immune response by recognizing m6A methylation (14). It is the m6A sign that contributes to the recognition of transcripts encoding lysosomal proteases by YTHDF1, and such appreciation of YTHDF1 enhances the abundance of lysosomal cathepsins in dendritic cells, eliciting the cross-presentation of these immune cells (15). The evidence that supports susceptibility to m6A regulation in the immune context has been accumulating extensively over the past years, while similar studies on AD are still scarce. A thorough investigation of the immune alterations and immune-inflammatory mechanism of m6A and AD, as well as the exact roles of m6A regulators in these changes, is, therefore, urgently required to shed light on the onset of AD from a brand-new aspect.

Herein, we surfed on the wave of the current state of knowledge on AD and systematically assessed the association between m6A regulators and the immune microenvironment (IME) features of AD by a series of bioinformatic analyses. Importantly, we investigated the diagnostic significance of m6A and IME in AD by assessing the efficacy of the AD diagnostic nomogram. FTO and IGF2BP1 mainly engaged in the IME modulation of the AD process in 21 m6A genes, and SYNC and MAPK1IP1L were also involved in AD with a high m6A level context. The diagnostic model integrating FTO, IGF2BP1, and MAPK1IP1L performed excellently in discriminating AD samples. Both the richness of infiltrating immune cell population and the genome alternations of immune response in AD have been remarkably connected with m6A regulators, indicating a close-knit link between m6A coordinators and immune monitors. Furthermore, there were distinct features of IME in AD subgroups defined by different m6A backgrounds, explaining their attractive diagnostic potential in clinical settings.

## Materials and methods

### Data collection and processing

Four AD-associated Gene Expression Omnibus datasets including GSE52093, GSE98770, GSE147026, GSE153434, and GSE107844 were selected for the following investigation. Transcriptome profiling data were downloaded from GEO (http://www.ncbi.nlm.nih.gov/geo/). We merged GSE52093, GSE98770, GSE147026, and GSE153434 and batch corrected them using the R package “sva” and used GSE107844 as the validation dataset. Baseline characteristics related to the datasets are listed in Supplementary Table 1.

### Differential analysis and correlation analysis

A total of 21 m6A-related genes including m6A writers such as METTL3, METTL14, WTAP, ZC3H13, and RBM15/15B; m6A erasers such as ALKBH5 and FTO; and m6A readers such as YTHDC1/2, YTHDF1/2/3, CBLL1, HNRNPC/A2B1, FMR1, LRPPRC, RBMX, ELAV1, and IGF2BP1 were extracted from expression profile data using the R software and investigated their expression levels between AD and the healthy group. Correlation analysis between 21 m6A genes and each other was implemented using the Spearman method. Correlation analysis of the immune cells and m6A genes was conducted using the Pearson correlation analysis.

### SsGSEA and XGboost analysis

We adopted the ssGSEA method in the Gene Set Variation Analysis (GSVA) package of R to probe the various immune cell infiltrating levels (16, 17). The XGboost algorithm was used to filter out the crucial m6A genes that contributed greatly to AD development.

### IME analysis in AD subgroups identified by 21 m6A gene expression profiles

The consensus cluster classifier algorithm was chosen to make a classification for AD based on the 21 m6A gene expression profile in AD samples, and the resulting cluster was validated using principal component analysis (PCA) (18, 19). Then, the 21 m6A gene expression level was examined in the two identified AD clusters. Also, IME feature analysis including immune response gene sets, HLA family genes, and immune cells between two novel AD subgroups was also explored.

### IME analysis in AD subgroups based on m6A scores *via* PCA analysis

First, PCA was used to calculate the m6A score, which divided AD samples into high and low m6A score groups according to the median m6A score. The IME ingredient differences between high and low m6A score groups such as immune response gene sets, HLA family genes, and immune cells were scrutinized.

### Identification of differentially expressed genes and functional enrichment analysis

The differentially expressed gene (DEG) list between high and low m6A score groups was obtained (filter rule: |LogFC|>1, *p* < 0.05), and the DEG list between AD and healthy samples (filter rule: |LogFC|>1, *p* < 0.05) was also collected at the same time. Then, we selected the shared common DEGs in two DEG lists using the Venn diagram and performed gene ontology (GO) and Kyoto Encyclopedia of Genes and Genomes (KEGG) enrichment analyses.

### AD diagnostic model construct

The random forest (RF) algorithm was used to pick out the predominant genes in common DEGs. Key genes and m6A key genes IGFBP2 and FTO were used to create a clinical diagnostic nomogram to determine the effect of these key factors on AD outcome. Later, calibration curves, DCA, and clinical impact curves were implemented to verify the efficacy of the diagnostic model. We subsequently verified the expression of these four genes using GSE107844.

## Result

### Expression pattern of m6A regulators in healthy and AD samples

**Figure 1:**
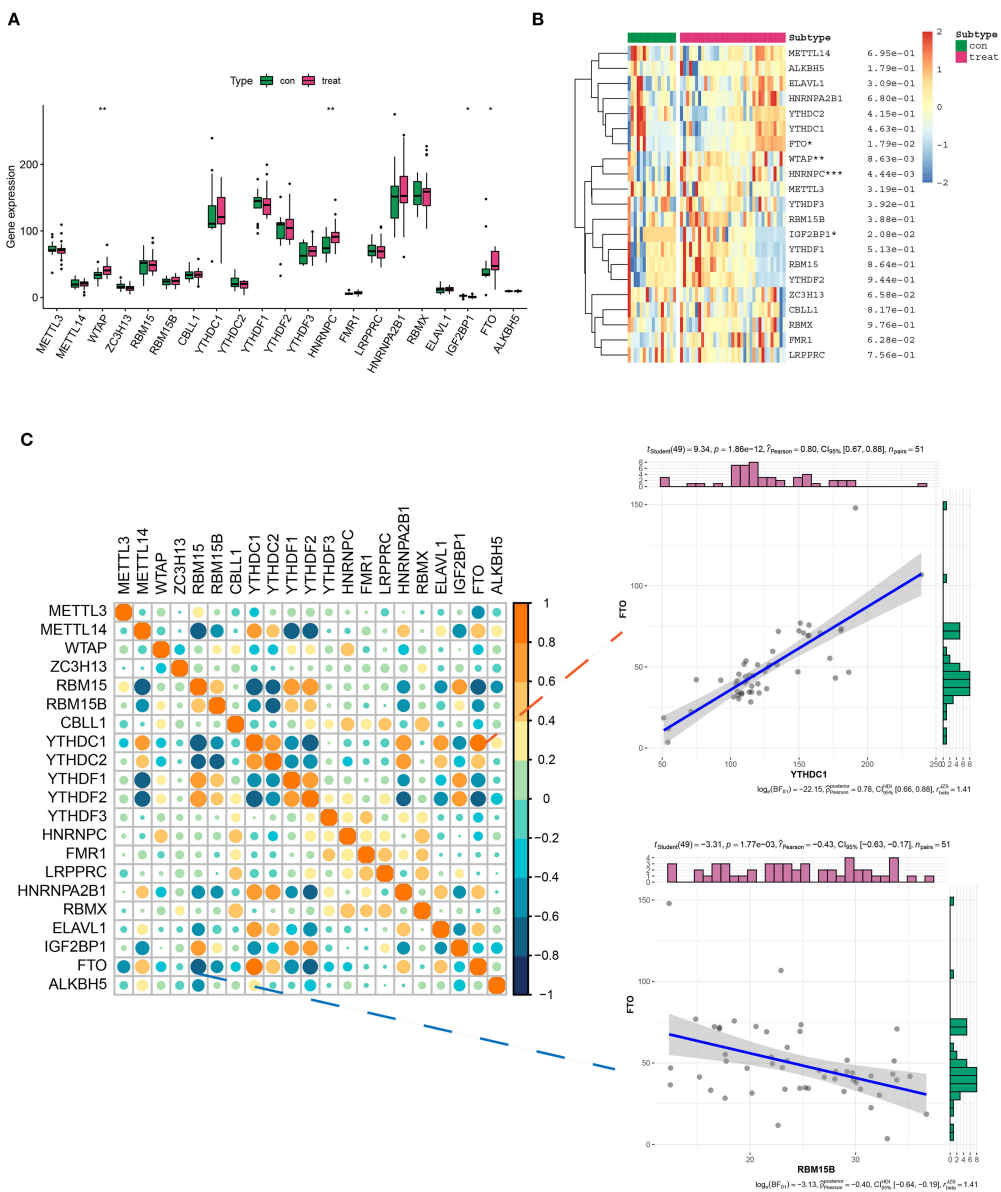
Differential expression landscape of 21 m6A genes in aortic dissection (AD). **(A,B)** The box plot and heatmap plot showed the expression level of 21 m6A genes in healthy and AD samples. **(C)** The correlation between the 21 m6A gene expressions in AD samples. In addition, two scatter plots showed two pairs of highly correlated m6A genes: positive correlation group (FTO and YTHDC1) and negative correlation group (FTO and RBM15B). *p < 0.05, **p < 0.01, ***p < 0.001 indicated the statistical significance of data.

The expression pattern of 21 m6A genes (m6A writers: METTL3, METTL14, WTAP, ZC3H13, and RBM15/15B; m6A erasers: ALKBH5 and FTO; and m6A readers: YTHDC1/2, YTHDF1/2/3, CBLL1, HNRNPC/A2B1, FMR1, LRPPRC, RBMX, ELAV1, and IGF2BP1) in healthy and AD samples was investigated in GSE52093, GSE98770, GSE147026, and GSE153434 datasets. The results showed that the expression levels of WTAP, HNRNPC, IGF2BP1, and FTO exhibited a striking distinction between healthy and AD samples, among which WTAP, HNRNPC, and FTO were significantly upregulated, while IGF2BP1 was downregulated compared with the normal group (Figures 1A,B). Intriguingly, there may exist a window in which these regulators can communicate and interact more actively and informatively, rather than in isolation (20, 21). Therefore, we investigated the correlation between 21 m6A genes in AD samples. Correspondingly, of all the correlations of m6A-managers, FTO was positively associated with YTHDC1 (*R* = 0.80), whereas negatively related to RBM15B (*R* = −0.48), suggesting that FTO may position at the center of the m6A evolution vortex in AD (Figure 1C).

### The association between m6A genes and immune infiltration in AD

**Figure 2:**
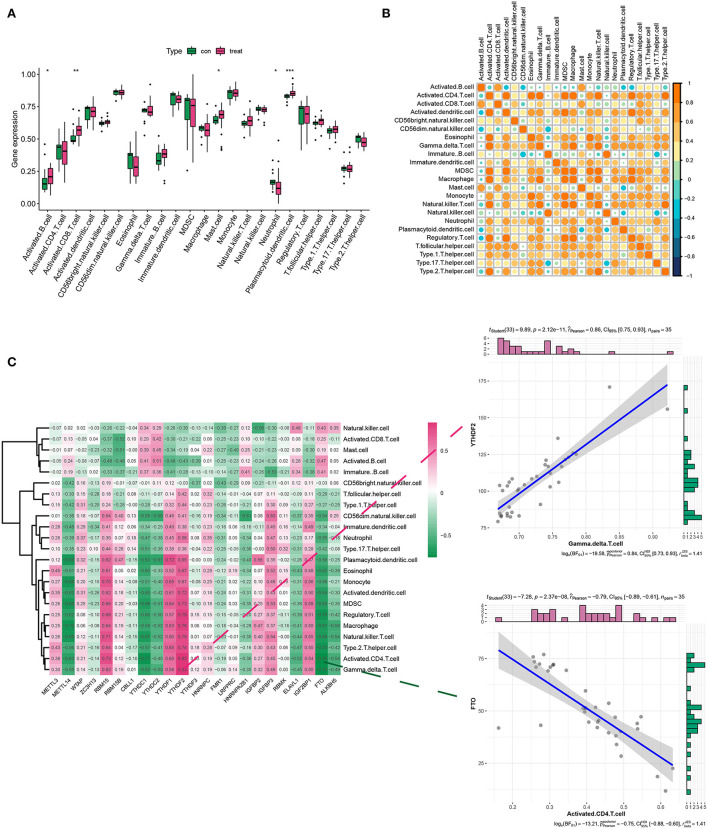
Analysis of immune infiltration in the healthy and AD groups. **(A,B)** The box plot and heatmap plot displayed various immune cell infiltration between the healthy and AD groups. **(C)** The correlation between m6A genes and immune infiltration cells in AD. The two scatter plots demonstrated the most positive related immunocyte-m6A regulator pair was YTHDF1-γδT cell, while the most negative immunocyte-m6A regulator pair was FTO-activated CD4+ T cell. *p < 0.05, **p < 0.01, ***p < 0.001 indicated the statistical significance of data.

Considering the role of immune response in AD conditions, we assessed the immune cell infiltration in AD *via* ssGSEA analysis. Results indicated that there was a differential expression of activated B cells, CD8+ T cells, mast cells, neutrophils, plasmacytoid, and dendritic cells (DCs) between the healthy and AD groups. Among them, activated B cells and CD8+T cells, plasmacytoid, and DCs showed a dramatic increase in their density (Figure 2A). Figure 2B describes the correlations of 23 immune cell types. Evidently, a positive correlation was more acceptable in AD. Then, we analyzed the association between 21 m6A regulators and immune cell abundance. Specifically, RBM15, YTHDF1, and YTHDF2 were positively associated with a large portion of infiltrating immune cells (Figure 2C). In contrast, METTL14, YTHDC1, YTHDC2, and FTO exerted the opposite properties (Figure 2C). More importantly, YTHDF1 showed the strongest positive interaction with γδ T cells, while FTO exhibited the most negative interrelationship with activated CD4+ T cells (Figure 2C).

### Identification of paramount m6A genes in AD based on XGboost

**Figure 3:**
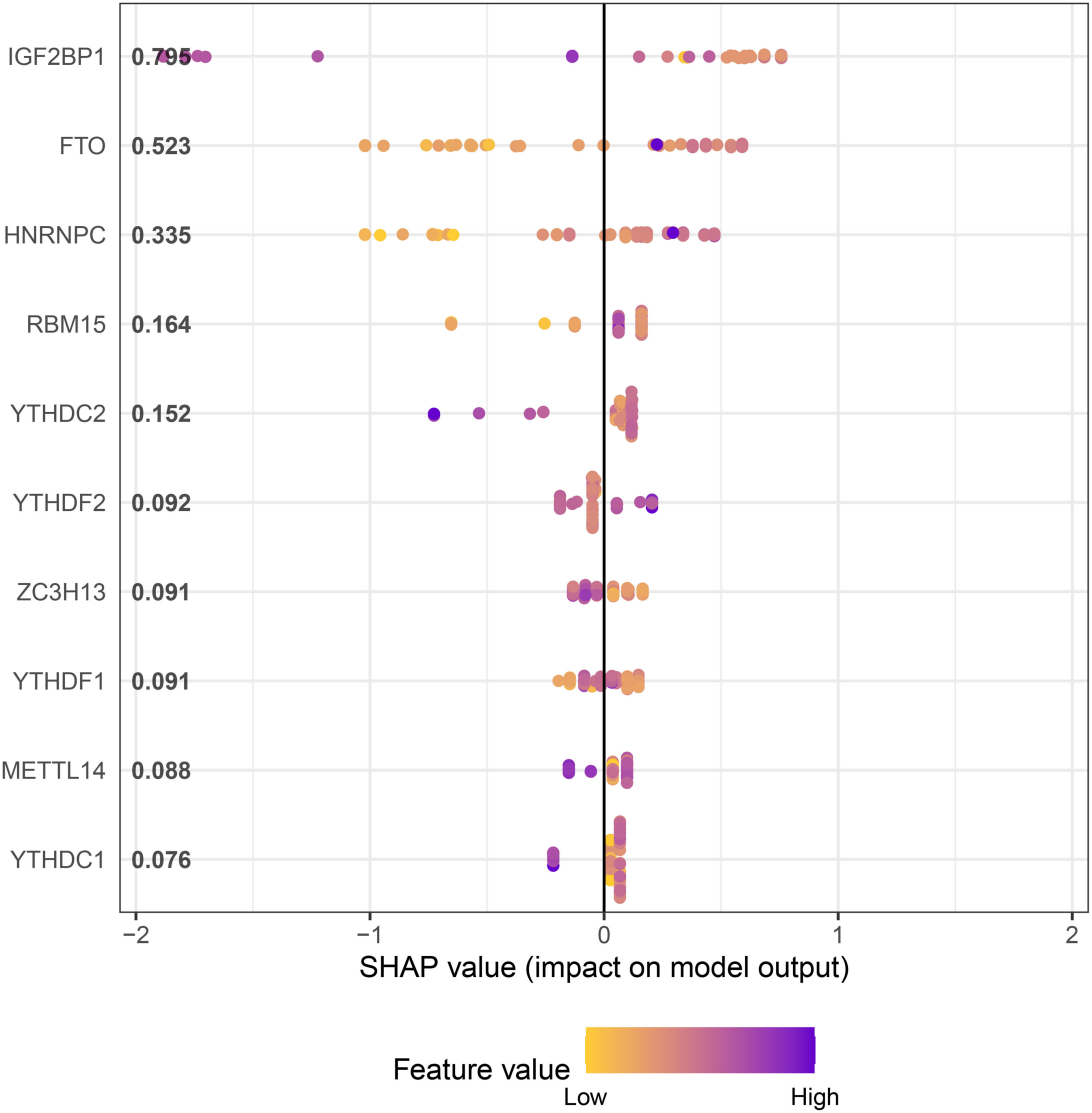
XGboost screens for m6A genes with a significant impact on AD.

**Figure 4:**
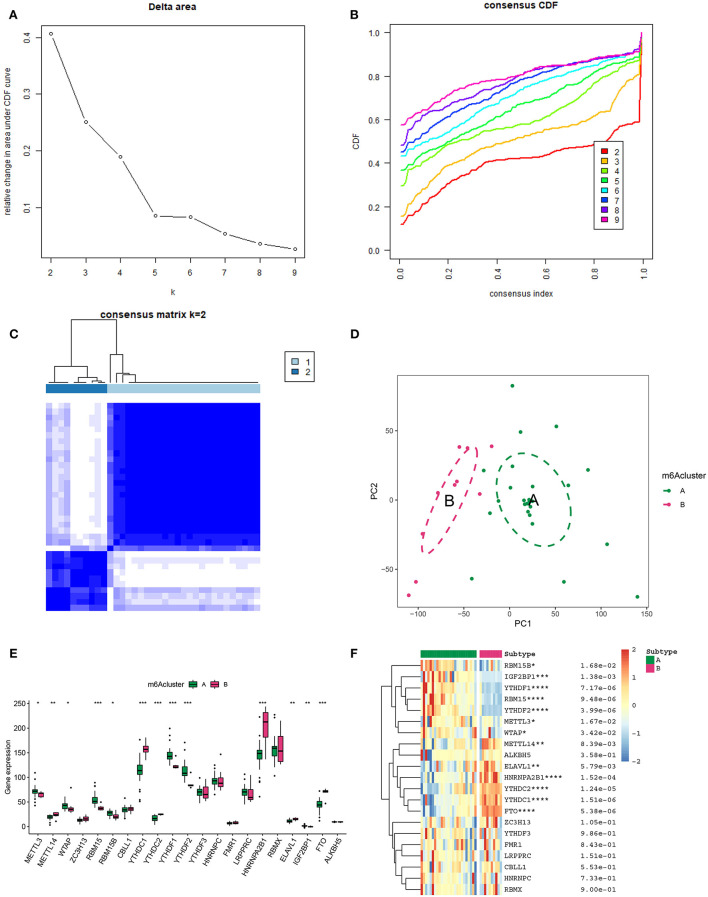
Unsupervised clustering of 21 m6A genes in AD samples. **(A)** Relative alterations in the area under the cumulative distribution function (CDF) curve for *k* = 2-9. **(B)** Consensus clustering CDF for *k*=2-9. **(C)** Heatmap of the co-occurrence ratio matrix of AD samples. **(D)** Principal component analysis of the transcriptome profiles of the 2 m6A clusters revealed significant differences in the transcriptomes of different m6A modification patterns. **(E)** The expression level of 21 m6A genes in two m6A AD clusters. **(F)** Unsupervised classifying of 21 m6A genes in two m6A AD clusters. *p < 0.05, **p < 0.01, ***p < 0.001 indicated the statistical significance of data.

In an attempt to determine the key m6A genes in AD, we chose to use XGboost characterized by screening out m6A genes that have a non-negligible impact on AD (22, 23). In addition, the SHAP values of IGF2BP1 and FTO were found to be higher than 0.5, suggesting that they exerted important roles in AD progression (Figure 3). Subsequently, 21 m6A genes were employed to classify AD into two main subgroups (namely, cluster A and cluster B) *via* consensus cluster (Figures 4A–C). The following PCA analysis demonstrated that 21 m6A regulatory genes could distinguish cluster A from cluster B (Figure 4D). Interestingly, most m6A regulatory genes, including IGF2BP1 and FTO, were ectopically expressed in two AD subtypes (Figures 4E,F).

### IME character in two AD clusters

**Figure 5:**
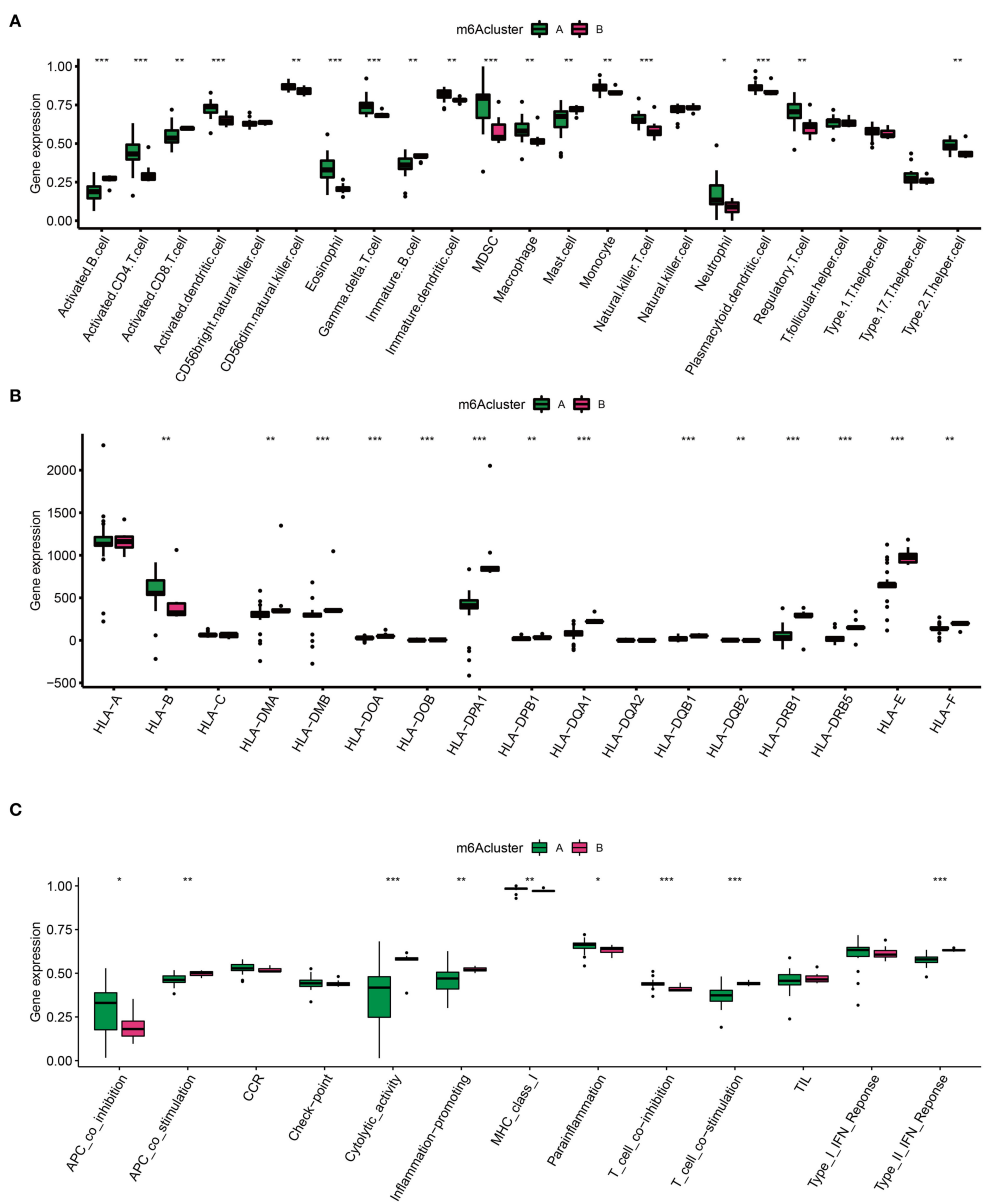
Immune microenvironment (IME) characteristics in two m6A AD clusters. **(A)** The immune cell infiltration status in two clusters. **(B)** The expression landscape of HLA family genes in two clusters. **(C)** The evaluation of immune response gene set in two clusters. *p < 0.05, **p < 0.01, ***p < 0.001 indicated the statistical significance of data.

Emerging data have found that a unique IME quality represents specific lesion types in different diseases (24). Considering the appealing indication capability of IME features, it is of great interest to detect IME traits in two AD clusters. To identify trends in IME characteristics between the two AD m6A clusters, we evaluated immune cell infiltration (Figure 5A), HLA family gene expression (Figure 5B), and immune response gene sets (Figure 5C). There was a high level of infiltrating immune cells, among which activated B cells, activated CD8+ T cells, and mast cells were significantly enriched in cluster B (Figure 5A). Simultaneously, the majority of HLA genes were upregulated in cluster B, and most immune genomics datasets were mainly enriched in cluster B (Figures 5B,C). These findings indicated that cluster B mediated a greater number of immune responses.

### The IME features in two AD m6A score clusters

**Figure 6:**
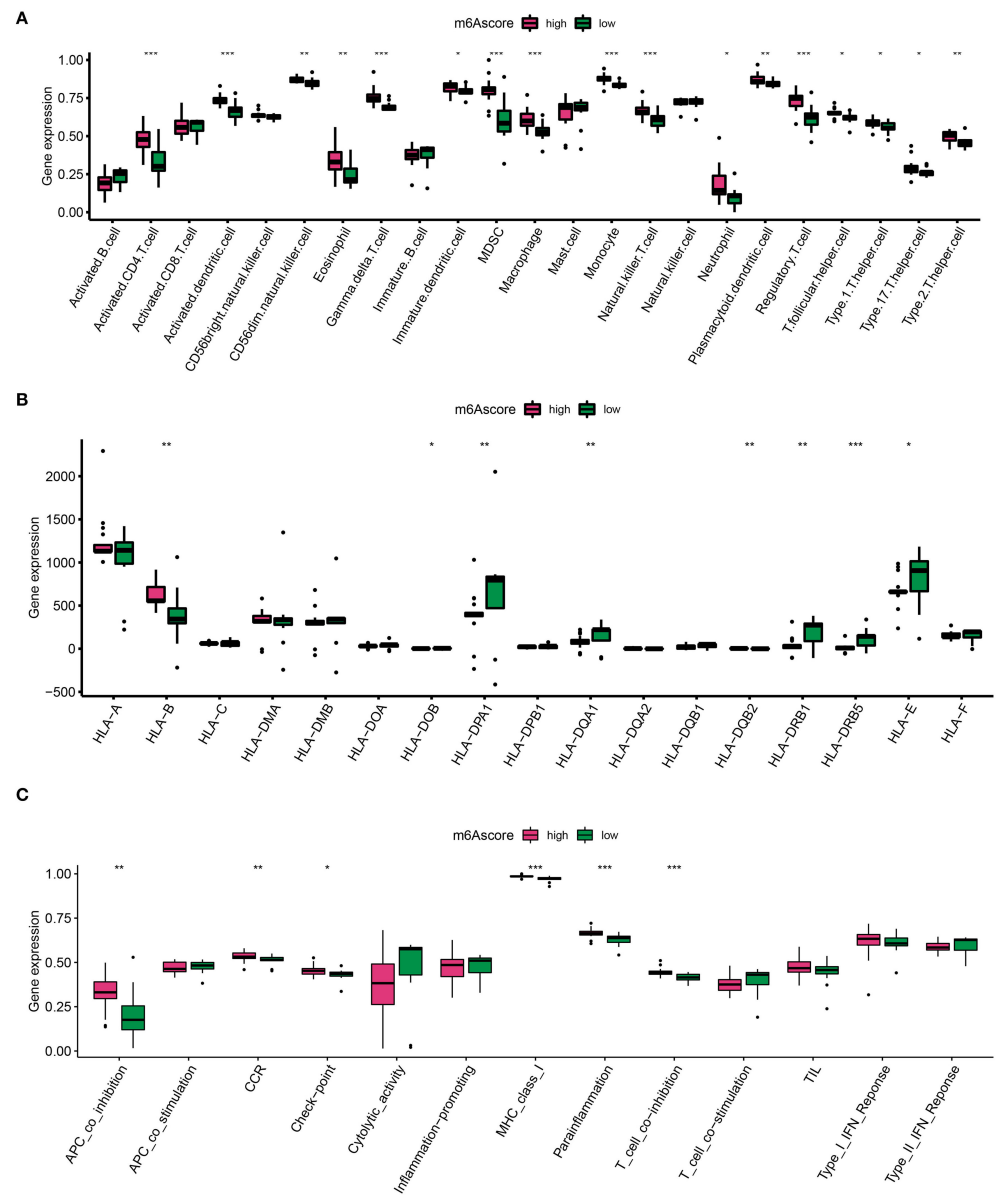
Diversity of IME characteristics of high and low m6A score groups. **(A)** The immune cell infiltration status in high and low m6A score groups. **(B)** The expression landscape of HLA family genes in high and low m6A score groups. **(C)** The evaluation of immune response gene set in high and low m6A score groups. *p < 0.05, **p < 0.01, ***p < 0.001 indicated the statistical significance of data.

The m6A score was calculated by PCA, which divided AD into high and low m6A score groups based on the score median. Similarly, to identify trends in IME ingredients between the two AD m6A score groups, we evaluated immune cell infiltration (Figure 6A), HLA family gene expression (Figure 6B), and immune response gene sets (Figure 6C) once again. A higher level of immune cell infiltration was detected in the high m6A score group, in which most immune cells were assembled and both HLA-A and HLA-B were overexpressed (Figures 6A,B). Furthermore, immunogenomic scores of APC co-inhibition, CCR, checkpoint, MHC-I, para-inflammation, and T cells exhibited superior immunogenomic scores in the high m6A score group (Figure 6C). These findings depicted that the high m6A score group was able to elicit a richer and more potent immune response.

### GO and KEGG analysis of DEGs in two AD m6A score groups

**Figure 7:**
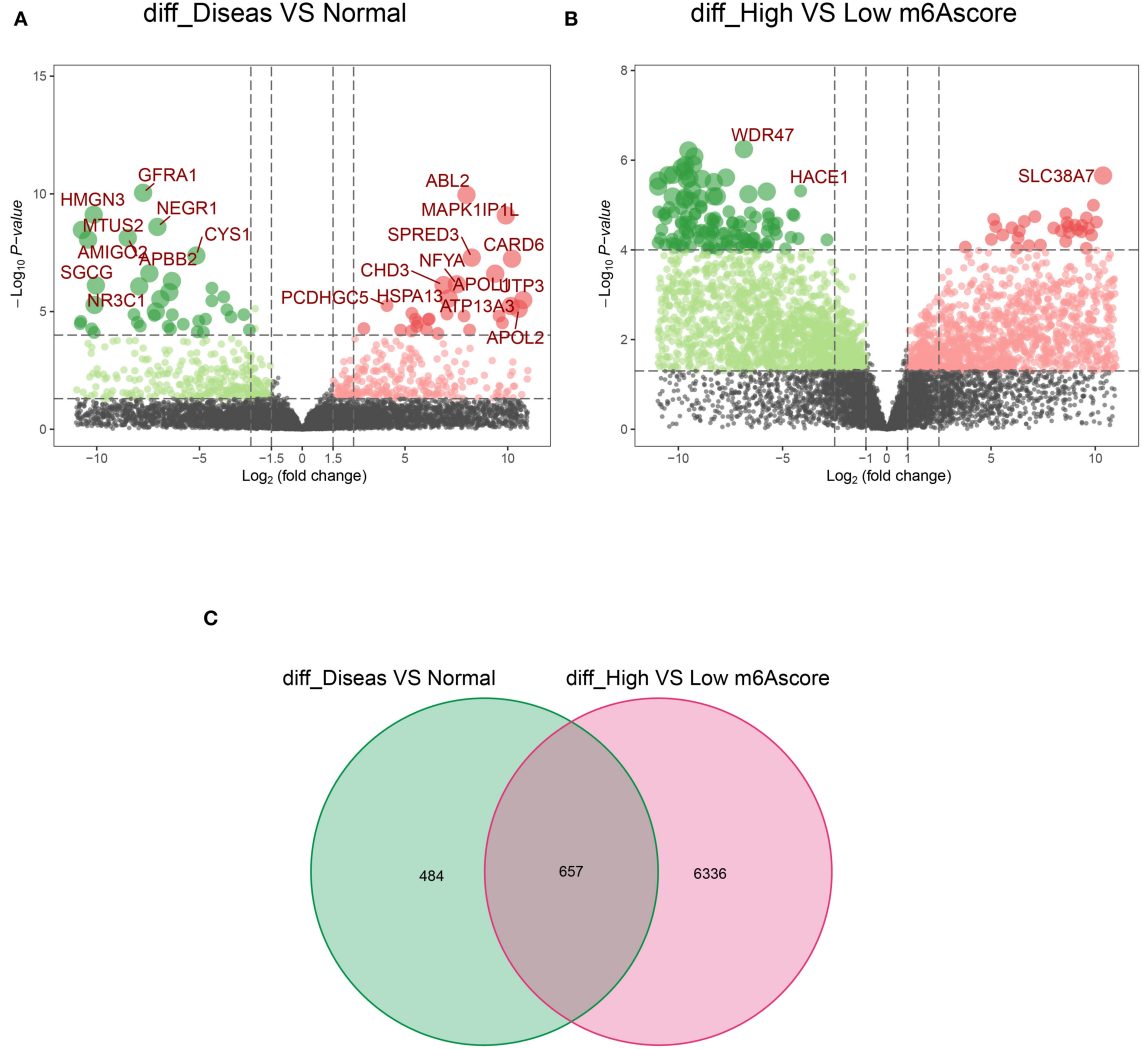
The acquisition of common 657 differentially expressed genes (DEGs). **(A)** The volcano plot of 1,141 DEGs in healthy and AD samples. **(B)** The volcano plot of 7,039 DEGs in the high score and low score m6A groups. **(C)** A total of 657 common DEGs from A and B in the Venn diagram.

**Figure 8:**
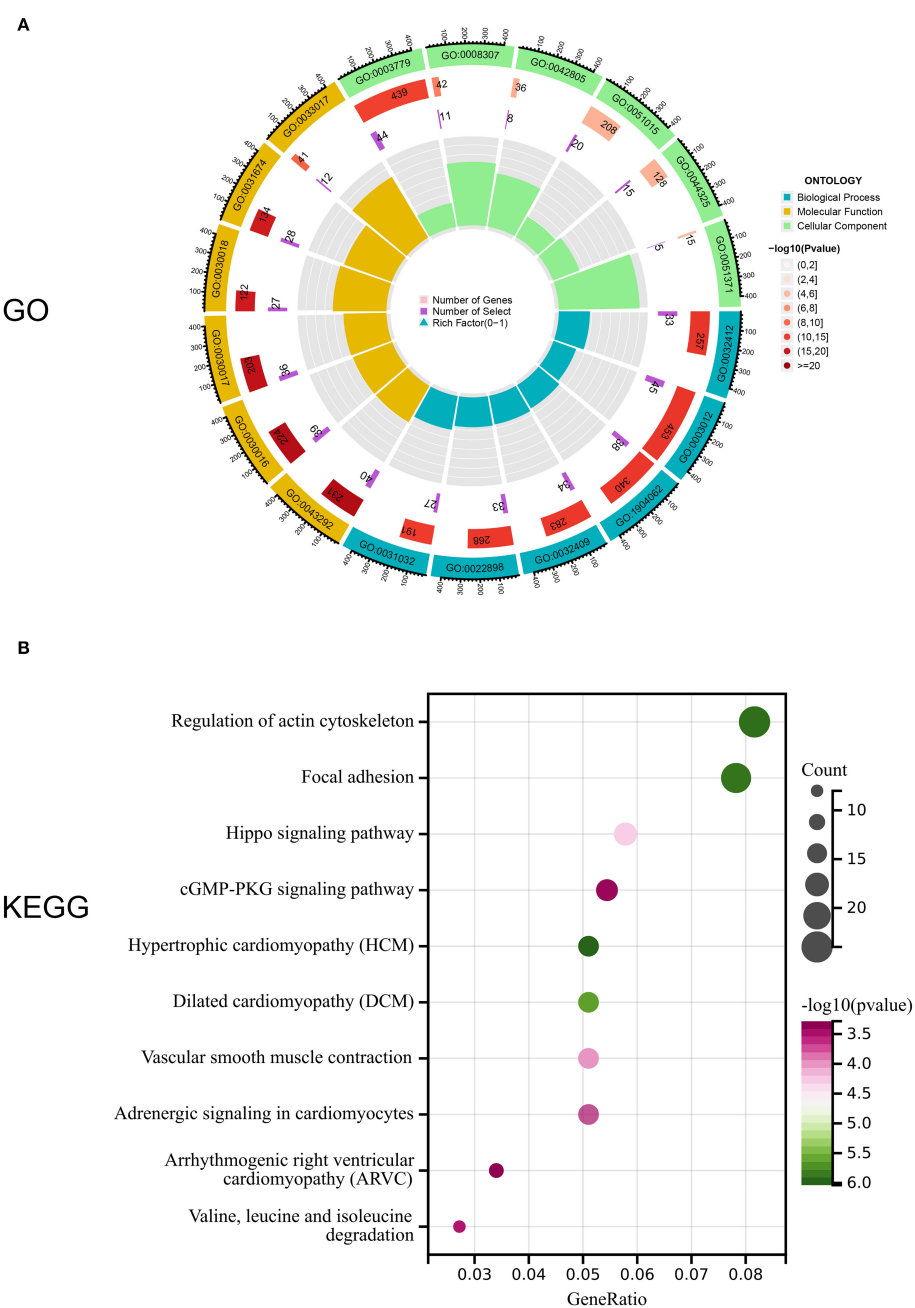
Enrichment analysis of common DEGs. **(A)** Gene ontology (GO) enrichment analysis of 657 common DEGs in biological functions (BFs), molecular functions (MFs), and cellular constitutions (CCs). **(B)** Kyoto Encyclopedia of Genes and Genomes (KEGG) enrichment analysis for 657,657 common DEGs.

Notably, 1,141 DEGs between high and low m6A score groups were obtained (|LogFC|>1, *p* < 0.05 Figure 7A), together with 7,039 DEGs between the AD and healthy groups (|LogFC|>1, *p* < 0.05 Figure 7B). A total of 657 common genes existing simultaneously in these two DEG lists simultaneously were selected for gene ontology (GO) and KEGG analysis (Figure 7C). GO results indicated that in biological functions (BFs), the ion transmembrane transporter activity and related regulation of muscle system process were remarkedly enriched; in terms of molecular functions (MFs), the structural constituent of muscle and actinin binding exhibited a significant enrichment. As for cellular constitutions (CCs), it was observed that contractile fiber and myofibril were abundant (Figure 8A, Table 1). Alternatively, the outcomes of KEGG analysis represented a dramatic enrichment in the regulation of actin cytoskeleton, hypertrophic cardiomyopathy, focal adhesion, dilated cardiomyopathy, and vascular smooth muscle contraction (Figure 8B). It followed that these DEGs were primarily associated with cardiac muscle system energy metabolism of cardiac muscle system as well as aberrant heart disease.

**Table 1:** Gene ontology (GO) pathway enrichment analysis.

**Ontology**	**ID**	**Description**	***P*-value**	***P*-adjust**	**Count**
BP	GO:0032412	Regulation of ion transmembrane transporter activity	1.07E-11	3.09E-08	33
BP	GO:0003012	Muscle system process	1.64E-11	3.09E-08	45
BP	GO:1904062	Regulation of cation transmembrane transport	2.06E-11	3.09E-08	38
BP	GO:0032409	Regulation of transporter activity	3.34E-11	3.09E-08	34
BP	GO:0022898	Regulation of transmembrane transporter activity	3.36E-11	3.09E-08	33
BP	GO:0031032	Actomyosin structure organization	9.10E-11	6.70E-08	27
CC	GO:0043292	Contractile fiber	3.33E-18	1.77E-15	40
CC	GO:0030016	Myofibril	7.28E-18	1.94E-15	39
CC	GO:0030017	Sarcomere	7.51E-17	1.33E-14	36
CC	GO:0030018	z disc	1.90E-15	2.53E-13	27
CC	GO:0031674	i band	2.76E-15	2.93E-13	28
CC	GO:0033017	Sarcoplasmic reticulum membrane	4.22E-09	3.74E-07	12
MF	GO:0003779	Actin binding	1.62E-10	1.24E-07	44
MF	GO:0008307	Structural constituent of muscle	1.08E-07	4.15E-05	11
MF	GO:0042805	Actinin binding	2.26E-05	0.004752	8
MF	GO:0051015	Actin filament binding	3.07E-05	0.004752	20
MF	GO:0044325	Ion channel binding	3.09E-05	0.004752	15
MF	GO:0051371	Muscle alpha-actinin binding	0.000102	0.013094	5

### Identification of key genes from 657 common DEGs by RF screening

**Figure 9:**
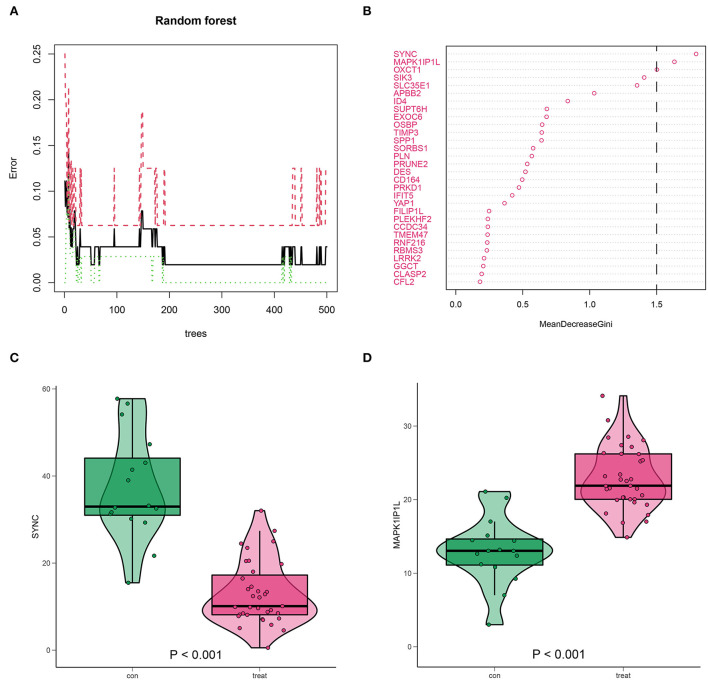
Identification for high m6A AD-associated key genes by random forest (RF). **(A)** RF screening for 657 DEGs. **(B)** SYNC and MAPK1IP1L (mean decrease Gini score >1.5) scored top in the gene importance score list. **(C,D)** The expression level of SYNC and MAPK1IP1L in AD samples.

The predominant genes of AD in 657 DEGs were picked out using the RF method (Figure 9A). These selected genes were ranked according to their mean decrease Gini, among which SYNC and MAPK1IP1L ranked top and were classified as crucial genes (Figure 9B). Notably, SYNC was downregulated in AD (Figure 9C), while MAPK1IP1L was upregulated in AD (Figure 9D), suggesting their attractiveness as prognostic biomarkers for AD with high m6A background. In the validation dataset GSE107844, SYNC was also less expressed in AD (Supplementary Figure 1).

### AD diagnostic model construct

**Figure 10:**
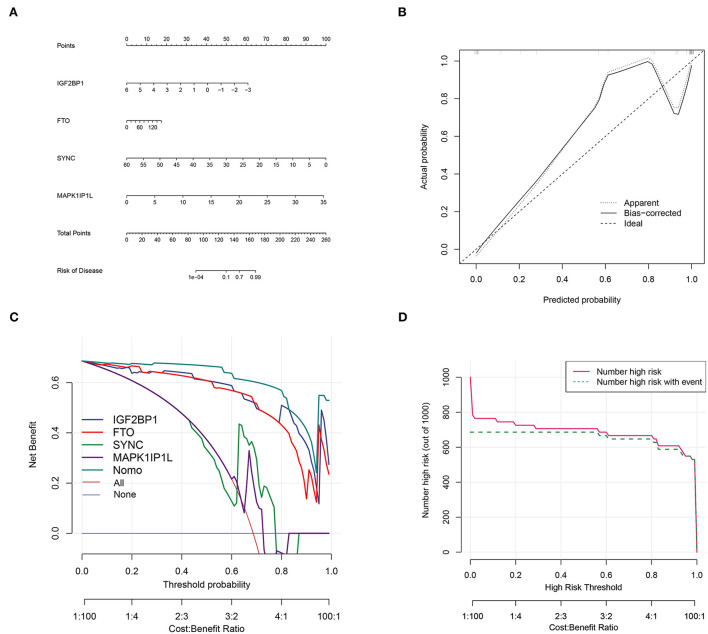
Aortic dissection diagnostic model construction based on four genes. **(A)** Construction of clinical diagnostic nomogram based on SYNC and MAPK1IP1L and IGF2BP1 and FTO. **(B)** The correction curve showed relatively high accuracy in predicting AD samples. **(C)** Decision curve analyses confirmed the benefit of this diagnostic model. **(D)** The clinical impact curve exhibited good predicated probability.

Top genes SYNC and MAPK1IP1L and dominant m6A genes IGF2BP1 and FTO (Figure 3) were selected to construct a clinical diagnostic model (Figure 10A). The calibration curve indicated that the SYNC/MAPK1IP1L/IGF2BP1/FTO model displayed a relatively excellent accuracy in predicting AD (Figure 10B). Additionally, the decision curve analysis also indicated that patients could benefit better from the constructed diagnostic model compared with individual factors (Figure 10C). In addition, the clinical impact curve confirmed the promising predictive ability of this diagnostic model (Figure 10D).

## Discussion

Recently, increasing attention has been paid to the role of m6A in AD. The MeRIP-seq and RNA-seq transcriptome data from AD samples revealed that the total m6A level of mRNA was high in the tissues of the ascending aortas of AD compared with corresponding normal tissues, implying that m6A was linked to AD progression. Furthermore, METTL14 methylated mRNA and eraser FTO demethylated mRNA, which are orchestrated with each other to deposit m6A remarks in mRNA (25). In this study, we found that a majority of m6A regulators were ectopically expressed in AD groups, uncovering that they largely participated in the development of AD. Specifically, WTAP, HNRNPC, and FTO were highly expressed in AD while IGF2BP1 was lowly expressed in AD. Taken together, it is conclusive that m6A exerted critical function in AD progression, while the underlying mechanism remains unclear. Of course, the cell assay data will make it more convincing.

Notably, FTO was found to upregulate and contribute to cell proliferation and migration in vascular smooth muscle cells (VSMCs), whose plastic phenotype conversion engaged in early AD (26). In this study, we found that FTO was identified as the crucial gene in IME modulation, implying that FTO promoted VSMC phenotype conversion by regulating the crosstalk with the immune cells in the microenvironment. Consistent with our findings, FTO was found to be aberrantly expressed in different inflammatory cell types, and it could coordinate T cell homeostasis through m6A methylation (27, 28). However, it seems that there was limited evidence focusing on the function of IGF2BP1 in AD. More research will be needed to clarify the association between IGF2BP1 and AD. Besides, we found a significant difference in activated B cell, activated CD8+T cell, mast cell, neutrophil, plasmacytoid, and dendritic cell abundance between AD and healthy groups. These results showed that m6A engaged in AD development by regulating immune cells.

Evidence is accumulating that m6A modification is responsible for the regulation of both innate and adaptive immune responses (29, 30). Most recently, the attention of the scientific community has gradually focused on the functions of m6A in immunity, in particular, tumor IME infiltrating cells, and the consequence has confirmed the multifaceted roles of m6A in cancer immunity (31, 32). Herein, we observed that the abnormally expressed YTHDF1 tended to positively represent the accumulation of unconventional Tγδ cells, whereas FTO was negatively related to activated CD4+ T cell aggregation in AD. Previous studies documented that the CD4+ T cell was tightly connected with vascular conditions, including atherosclerosis, hypertension, and AD (33). There were a large number of infiltrating inflammatory cells, including various CD4+ T cells and macrophages, which have been demonstrated to be involved in inflammatory responses through secreting functional cytokines or cross-talking with other immune cells, thus participating in AD (34, 35). Therefore, we speculated that FTO engaged in AD development by regulating CD4+ T cell function.

Aortic dissection cluster B identified by the m6A gene pattern had a higher level of immune cell infiltration such as activated B cells, activated CD8+T cells, and mast cells, as well as high HLA genes and immune gene expression levels. These results suggest that cluster B mediated more immune responses and contributed greatly to AD inflammation. In addition, we divided AD samples into high m6A level and low m6A score groups. Certainly, immense immune cell infiltration and remarkable expression of HLA-A and HLA-B were associated with high m6A levels. Additionally, the APC co-inhibition, CCR, check-point, MHC-class-I, parainflammation, and T cell co-inhibition immune genes were expressed strongly in the high m6A score group. These findings illustrate that a high m6A modification level epigenetic background mediates immune cell infiltration AD inflammation.

Furthermore, GO and KEGG results from shared DEGs in both AD vs. healthy and high m6A score vs. low group indicated that they were mainly participating in the energy metabolism of the cardiac muscle system and aberrant heart diseases, which was consistent with the concept of the clinical symptoms of AD focused on the cardiac system. Subsequently, we screened out key genes (SYNC and MAPK1IP1L) by RF analysis from the common DEGs. Furthermore, we integrated SYNC and MAPK1IP1L and crucial m6A regulators IGF2BP1 and FTO to establish a clinical diagnostic model for AD. Interestingly, the classifier built performs well in distinguishing AD samples, further confirming the pivotal role of the m6A moderator in AD. Taken together, our findings certainly demonstrate that m6A modifications exert a significant impact on the immune context of AD and provide new insights to delineate the pathogenesis and underlying mechanisms of AD.

## Conclusion

Our study elucidated that m6A modification makes great contributions to the consolidation of the AD immune contexture. FTO and IGF2BP1 were identified as the predominant m6A genes in remodeling IME of AD, and SYNC and MAPK1IP1L were verified as the key genes in AD with a high m6A level context.

## Data availability statement

The original contributions presented in the study are included in the article/Supplementary material, further inquiries can be directed to the corresponding author.

## Author contributions

All authors listed have made a substantial, direct, and intellectual contribution to the work and approved it for publication.

## Funding

This study was supported by grants from the Scientific and Technological Project of Zhengzhou University (212102310169).

## Conflict of interest

The authors declare that the research was conducted in the absence of any commercial or financial relationships that could be construed as a potential conflict of interest.

## Publisher's note

All claims expressed in this article are solely those of the authors and do not necessarily represent those of their affiliated organizations, or those of the publisher, the editors and the reviewers. Any product that may be evaluated in this article, or claim that may be made by its manufacturer, is not guaranteed or endorsed by the publisher.

## Supplementary material

Supplementary Figure 1 Expression of SYNC, MAPK1IP1L, IGF2BP1, and FTO in GSE107844.
