# Supplementary figures and images for: The diagnostic significance of integrating m6A modification and immune microenvironment features based on bioinformatic investigation in aortic dissection
Source: Front Cardiovasc Med. 2022 Aug 29;9:948002. doi: 10.3389/fcvm.2022.948002 (PMC9464924; doi:10.3389/fcvm.2022.948002)

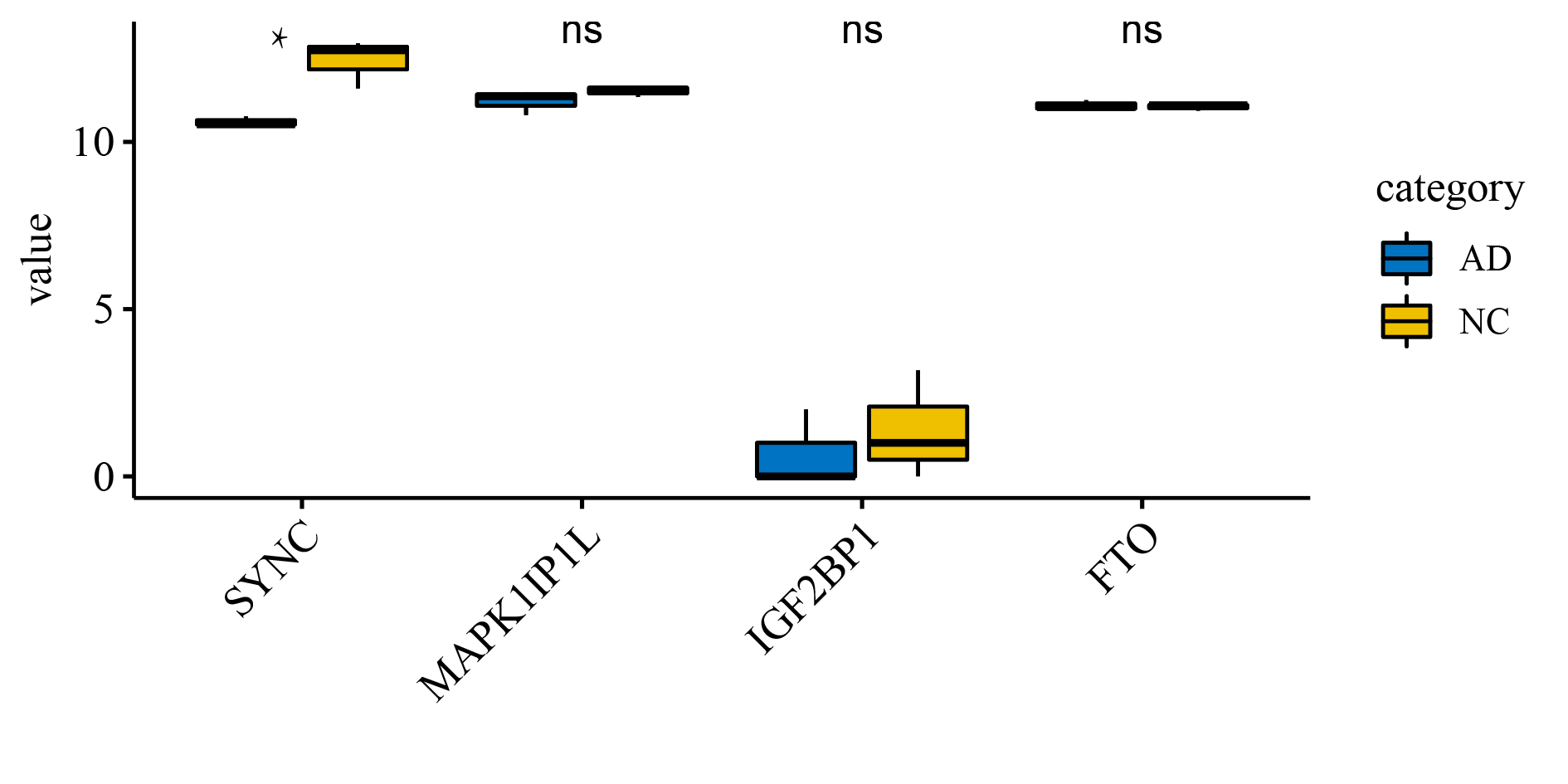

Supplement: Supplementary Figure 1 — Expression of SYNC, MAPK1IP1L, IGF2BP1, and FTO in GSE107844. [file Image_1.TIF]
