# Supplementary material for: The diagnostic significance of integrating m6A modification and immune microenvironment features based on bioinformatic investigation in aortic dissection
Source: Front Cardiovasc Med. 2022 Aug 29;9:948002. doi: 10.3389/fcvm.2022.948002 (PMC9464924; doi:10.3389/fcvm.2022.948002)
Supplement: Supplementary file 2 [file Table_1.docx]

Supplementary Table 1 Baseline characteristics related to the datasets

| Data | GSE52093 |  | GSE98770 | GSE147026 | GSE153434 | GSE107844 |
| --- | --- | --- | --- | --- | --- | --- |
| Cohort description | acute aortic dissection |  | aortic dissection | aortic dissection | aortic dissection | aortic dissection |
| Control (n) | 5 |  | 5 | 4 | 10 | 3 |
| Aortic dissection(n) | 7 |  | 6 | 4 | 10 | 3 |
| Platform | GPL10558 |  | GPL14550/  GPL17660 | GPL24676 | GPL20795 | GPL20301 |
| Organism | Homo sapiens |  | Homo sapiens | Homo sapiens | Homo sapiens | Homo sapiens |
| Male (n) | 10 |  | 8 | 0 | 0 | 5 |
| Female (n) | 1 |  | 3 | 0 | 0 | 1 |
